# Editorial: Fertilization and early development: genetics and epigenetics

**DOI:** 10.3389/fgene.2024.1465510

**Published:** 2024-07-30

**Authors:** Yu Tian, Yu-fan Wang, Yi-liang Miao, Li-quan Zhou

**Affiliations:** ^1^ Institute of Reproductive Health/Center of Reproductive Medicine, Tongji Medical College, Huazhong University of Science and Technology, Wuhan, Hubei, China; ^2^ Institute of Stem Cell and Regenerative Biology, College of Animal Science and Veterinary Medicine, Huazhong Agricultural University, Wuhan, Hubei, China

**Keywords:** germ cell, fertilization, early development, genetics, epigenetics

Fertilization and early development are processes characterized by delicate genetic and epigenetic regulation. In brief, sperm fuses with the oocyte to form a zygote, which then undergoes zygotic genome activation (ZGA) and lineage specification, ultimately implanting during the blastocyst stage. This process is intricately regulated by numerous key regulatory genes and critical epigenetic modifications, which are vital for the post-implantation development and subsequent pregnancy. This Research Topic comprises three original studies, one case report, and two reviews, which will advance our understanding of genetics and epigenetics in the fertilization and early embryonic development.

Germ cell maturation and embryo development require the precise gene expression program at different developmental stage, which sculpts the dynamic epigenetic modification landscape. Polycomb group (PcG) complex, with its two components PRC1 and PRC2, catalyzes the H2AK119ub1 and H3K27me3, which regulate cell fate by repressing gene expression. Li et al. reviewed the role of the PcG complex in mammalian development, contributing to our understanding of multifaceted functions of the PcG complex and H3K27me3. Additionally, H3K27me3 may be responsible for marking recurrent double-strand breaks (DSBs) in transgenerational DNA repair. Due to the absence of sister chromatids, the genome of haploid round spermatids cannot repair DSBs through homologous recombination repair (Kitaoka and Yamashita, 2024). How spermatids cope with this vulnerable genome state remains not fully elucidated. Scheuren et al. focused on the distribution of DSBs in human sperm, revealing a strong colocalization between H3K27me3 and recurrent DSBs. This result suggests the paternal H3K27me3 may serve as a guiding marker for maternal Polθ in the zygote to execute transgenerational DNA repair at sites of recurrent DSBs.

Following meiosis, round spermatids undergo intricate morphological changes to form mature spermatozoa. Abnormal sperm structures could prevent sperm from approaching or fusing with the oocyte, which is a key cause of fertilization failure. With the application of whole-genome sequencing, many genes associated with teratozoospermia have been identified. Bragina et al. analyzed samples from 12 globozoospermia patients and detected homozygous variants in *Dpy19l2* and *Spata16* in three of these cases. Mutations in *Dpy19l2* and *Spata16* have been identified as key causes of globozoospermia (Dam et al., 2007; Koscinski et al., 2011). Moreover, globozoospermia phenotype has also been validated in mouse with *Dpy19l2* or *Spata16* deficiency (Fujihara et al., 2017; Castaneda et al., 2021). Genetically engineered mouse models offer a pathway for studying the mechanisms of genetic diseases. However, because of the complexity of gene mutation and homology differences, mouse models may not always accurately mimic human phenotypes. Nguyen et al. conducted loss-of-function studies in mouse model on 13 testis-enriched genes including *Adam20* (A gene associated with fertilization failure in human), demonstrating that these genes are not essential for male fertility in mice. Therefore, we should cautiously evaluate the results derived from mouse models.

Currently, assisted reproductive technologies are widely used to address fertilization failure and to prevent the transmission of pathogenic parental genes to offspring (Brezina and Kutteh, 2015). Hu et al. firstly reported a woman with Hereditary Leiomyomatosis and Renal Cell Cancer syndrome who successfully delivered a healthy baby by preimplantation genetic testing for monogenic disorders (PGT-M). This case highlights the potential of PGT-M in addressing the reproductive needs of patients with genetic diseases. Apart from genetic defects, environmental pollutants can indirectly impair fertility by affecting gene expression and epigenetics (Green et al., 2021). Wang et al. discussed the reproductive toxicity of endocrine-disrupting chemicals (EDCs) in female reproduction, summarizing the current epidemiological studies and animal model studies for five major EDCs. Environmental pollutants can induce epigenetic alterations through oxidative stress and DNA damage, leading to impaired gene regulation and organelles dysfunction in germ cell or embryo (Strazzullo and Matarazzo, 2017; Lopez-Rodriguez et al., 2021). Therefore, healthy populations without genetic defects should also pay attention to the potential reproductive risks posed by environmental pollution.

Finally, we thank all authors for their contributions in the Research Topic. This Research Topic focuses on the genetic and epigenetic regulation of fertilization and early development, a field that has rapidly advanced over the recent years. While the scope of this Research Topic is limited, the progress it covers is exhilarating, as the advances in basic science are indeed contributing to the disease prevention and clinical intervention in reproductive medicine.
